# Many paths, similar destinations: viruses and bacterial microcompartments form polyhedra inside cells

**DOI:** 10.1128/jvi.01577-25

**Published:** 2026-04-20

**Authors:** Kristin N. Parent, Cheryl A. Kerfeld

**Affiliations:** 1 Department of Biochemistry and Molecular Biology, Michigan State University 3078, East Lansing, Michigan, USA; 2 Environmental Genomics and Systems Biology Division, Lawrence Berkeley National Laboratory 1666, Berkeley, California, USA; 3 MSU-DOE Plant Research Laboratory, Michigan State University 3078, East Lansing, Michigan, USA; 4 Molecular Biophysics and Integrated Bioimaging Division, Lawrence Berkeley National Laboratory 1666, Berkeley, California, USA; Indiana University Bloomington, Bloomington, Indiana, USA

**Keywords:** bacteriophage, virus, carboxysome, encapsulins, bacterial microcompartments

## Abstract

A large number of biological entities assemble into icosahedral structures, and these are ubiquitous throughout nature. Examples include eukaryotic and prokaryotic viral capsids and more recently discovered bacterial microcompartments. Viral capsids and bacterial microcompartments are both composed of pentameric and hexameric subunits; however, they differ in the type of cargo they encapsulate: nucleic acid or protein. Also, both depart from strict icosahedral symmetry: while this is less common in viruses, among bacterial microcompartments, diverse and heterogeneous polyhedra are common. We review shared principles and key distinctions between the self-directed assembly of various icosahedral architectures and their polyhedral variants in nature and explore the concept that there are multiple paths, influenced by their cargo, to arriving at similar protein cage morphologies.

## INTRODUCTION

Icosahedral proteinaceous shells are important containers for many biological functions. These shells are capable of self-directed assembly from one or a few subunits, are relatively optimal in surface to volume ratio, and are inherently stable and robust to protect their internal cargo. Understanding shell properties is crucial for a number of disciplines. In virology, many capsids are icosahedral and protect the genetic cargo from environmental onslaughts and host defense mechanisms. Understanding the structure and assembly pathways of viral shells can guide rational drug design, vaccine development, receptor or immune-antibody-binding studies, etc. (1, 2). For bacterial microcompartments (BMCs), these shells both cluster and protect segments of both anabolic (carboxysome) and catabolic (metabolosome) pathways. BMC shells also provide selective permeability for the exchange of metabolites with the bulk environment. Encapsulins are classified as bacterial nanocompartments and are distinct from BMCs. Encapsulins serve diverse functions, with roles in stress response, iron storage, and detoxification, to name a few (3, 4). Understanding BMC and encapsulin structures can inform us about the diverse functions of these shells in nature, and this knowledge can be used to guide the design of synthetic BMCs for bioengineering (5) and biomedical (6) applications.

### Scalability of shells: how to build bigger icosahedra and triangulation number

An icosahedron is simply a spherical structure with 12 vertices and 20 angular facets. Consider the classic organization of a soccer ball, with the black pentagonal shapes and the white hexagonal shapes. The simplest of these structures can be built from 60 subunits into a so-called Triangulation number 1 (“T=1”) assembly consisting entirely of pentagonal shapes. Bigger structures can be assembled by the addition of hexagonal shapes progressively into T=3, T=4, T=7….T=52 complexes, etc., according to this equation: T=*h^2^+hk+k^2^* (7). In this relationship, *h* and *k* define the complexity of the icosahedron and can be used to index the structure. The structure can be described as taking *h* steps away from a penton vertex and then taking *k* steps to get to the next fivefold vertex. In biology, pentagonal and hexagonal shapes are most often formed from individual protein chains that assemble into pentameric oligomers (five protein subunits) and hexameric oligomers (six protein subunits) used to build icosahedral particles. The T number can also be described as a multiple of 60 protein subunits (e.g., a T=3 capsid has 60*3, or 180 subunits, and a T=4 capsid has 60*4, or 240 units, etc.).

Bacteriophages, eukaryotic viruses (8), and bacterial microcompartments (9, 10) are examples of natural protein cages that can be formed with icosahedral symmetry but also depart from perfect symmetry to form other polyhedra. In this review, we will focus on the protein subunits, assembly pathways, and principles of forming biological shells, mainly focusing on viruses and bacterial microcompartments.

## THE BUILDING BLOCKS OF THE SHELLS OF VIRUSES AND BACTERIAL MICROCOMPARTMENTS

### Common structures: shell building blocks use a minimal number of folds

**Fig 1:**
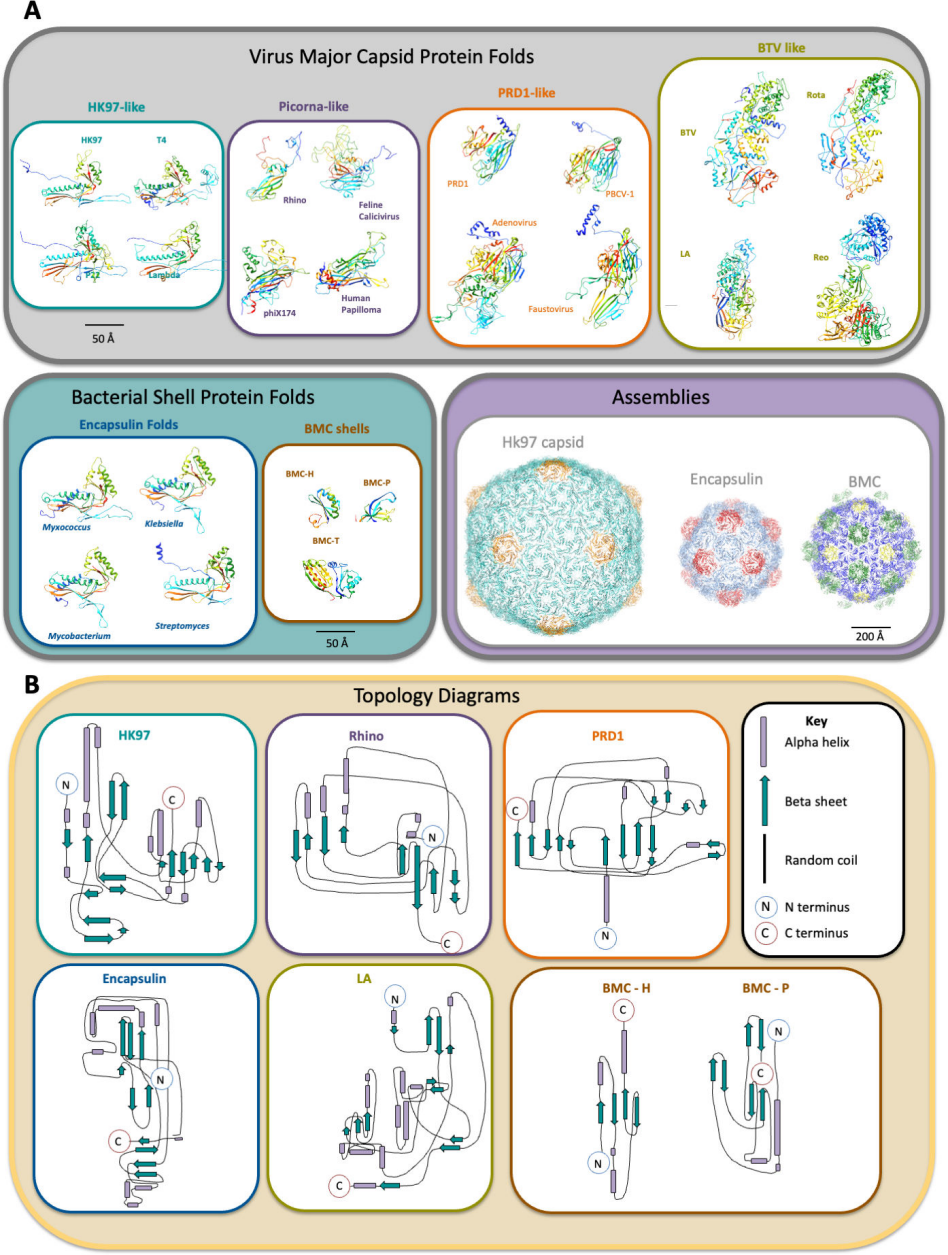
Structural comparison of a variety of biological icosahedra: Viruses and bacterial shells. (**A**) Examples of monomers from viruses are grouped according to the four known viral folds. Each monomer is colored from the N-terminus (blue) to the C-terminus (red). Both HK97 and encapsulins share a homologous fold, whereas the BMC shell proteins are unrelated. The HK97 fold group includes all known dsDNA-containing tailed phages and herpesviruses. Representatives are bacteriophages HK97 (PDB 1OHG), Phage T4 (PDB 1YUE), Phage P22 (PDB 8I1T), and Phage Lambda (PDB 7VIK). The picornavirus group includes small ssRNA and small ssDNA viruses such as rhinovirus-B5 (PDB 6SK6), Feline Calicivirus (PDB 3M8L), *Microviridae* member phage phiX174 (PDB 9K3M), and human papillomavirus (PDB 2R5K). The PRD1-like group spans larger dsDNA viruses such as adenoviruses, tailless dsDNA containing icosahedral phages, and giant viruses. Representatives are Phage PRD1 (PDB 1CJD), Cholovirus PBCV-1 (PDB 5TIP), adenovirus (PDB 1P30), and giant virus Fausovirus (PDB 5J7O). The Bluetongue virus group (BTV-like) includes dsRNA viruses such as BTV (PDB 2BTV), rotavirus (PDB 3KZ4), L-A virus (PDB 1M1C), and reovirus (PDB 1EJ6). Examples of monomers from a variety of encapsulins, including *Myxococcus xanthus* EncA (PDB 8VJO), *Klebsiella pneumoniae* DyP peroxidase (PDB 8U50), *Mycobacterium smegmatis* encapsulating shell (PDB 7BOJ), and *Streptomyces lydicus* Encapsulin 1 (PDB 9BJE): note the structural homology to the viral proteins in the HK97-like group. By comparison, bacterial microcompartment shell protein monomers have a distinct topology. The structures of BMC-H (hexamer), BMC-P (pentamer), and BMC-T (trimer) proteins from *Haliangium ochraceum* (HO) microcompartment shells (PDB 5V74). Assemblies of these monomers into icosahedral shells are also shown for HK97, *M. xanthus encapsulin*, and HO shells. Hexons and pentons are rendered in different colors. (**B**) Topology diagrams of a representative protein from each cluster of folds, generated with Pro-Origami (17).

The vast majority of viruses are icosahedra, formed from chemically identical protein subunits that can adopt different local configurations to accommodate pentameric and hexameric oligomerization according to the Caspar Klug theory of quasi equivalence (7). This has been extensively studied using a number of model systems. The first high-resolution structure of a bacteriophage capsid was from X-ray crystallography studies on the Hong Kong 97 phage capsid (11, 12). This has since become the namesake of this protein family (i.e., the “HK97 fold”). Proteins with the HK97 fold are not the only ones capable of building icosahedral structures in virology. There are currently four known common folds that drive capsid assembly in viral systems that span across domains of life, including examples within bacteria, archaea, and eukaryota (13), and each viral family has a shared fold among their members (Fig. 1). These four folds encompass (i) the HK97 fold across all known dsDNA containing tailed bacteriophages and the herpesviruses, (ii) the Bluetongue virus-like fold (“BTV-like”) common among dsRNA viruses, (iii) the PRD1/adenovirus lineage (“PRD1-like”) that spans larger dsDNA viruses such as adenoviruses and giant viruses, as well as some phages, and the (4) picornovirus-like fold for small ssRNA viruses, dsDNA such as SV40 and papillomavirus, and ssDNA viruses such as parvoviruses (13–16).

### Forming icosahedra from limited subunit types: efficient use of genome space

Studies on related phages such as bacteriophage P22 have also shed light on capsid assembly (18, 19). Phages like HK97 and P22 have ~40 kb genomes, and their capsids assemble into T=7 isometric cages (of ~50 nm diameter) from a single gene product called the major coat protein. Using the same building block for both hexons and pentons makes efficient use of genomic space since smaller viruses can only package a finite amount of genomic material. Rarely, some phages and viruses assemble from chemically distinct protein subunits derived from the expression of multiple genes. In these cases, one type of protein forms oligomers with cyclic fivefold symmetry called pentons, while a second protein forms cyclic oligomers with sixfold symmetry called hexons. Bacteriophage T4 falls into this category, where the hexons are formed from gp23 protein while the pentons are formed from gp24 protein (20). By comparison to HK97 or P22, phage T4 has a much larger genome (~145 kbp), a larger capsid organized into a prolate T=13 geometry, and a capsid size of 120 nm by 86 nm (21). Phage T4 can therefore more easily accommodate multiple genes encoding different capsid proteins. Another way of maximizing the efficiency of genomic space is achieved by eukaryotic viruses that assemble from two or three subunit types created from a single gene by either post-translational modifications, such as proteolytic cleavage seen in the processing of VP0 in polio (22, 23), or via translational regulation (e.g., alternative start sites observed in systems such as adeno-associated viruses [24] and polyomaviruses [25]).

While viral pentons and hexons can form from either chemically identical monomers in a quasi-equivalent conformation (most phages and herpesviruses), or from distinct protein subunits (such as phage T4, polio, and adeno-associated viruses, etc.), in general, the core unit of the monomers retains the same overall fold. For example, the HK97-fold is ubiquitous among all known dsDNA-containing bacteriophage and herpesviruses and is quite versatile for assembly into a large array of capsid sizes ranging in sizes from 50 to 180 nm diameters while maintaining the rules of icosahedral symmetry (26, 27).

Similarly, subcellular encapsulins form nanocages to compartmentalize biochemical reactions from chemically and structurally identical subunits (28). Two examples include the iron-storing encapsulin in *Myxococcus xanthus* (29)*,* and peroxidase encapsulins in enterobacteria (30). These encapsulins form cages from bacterially encoded proteins that are structurally homologous to the phage HK97 major coat protein (Fig. 1). There are now thousands of bioinformatically identified encapsulin systems across bacteria and archaea; all maintain the HK97 fold as their building blocks (31). There is a strong evolutionary relationship between encapsulins and bacteriophages, likely acquiring the same protein fold through horizontal gene transfer (32, 33). Generally, encapsulin assembly is high fidelity in native systems, but heterogeneous structure deviations occur when cargo loading is greatly enhanced (34).

**Fig 2:**
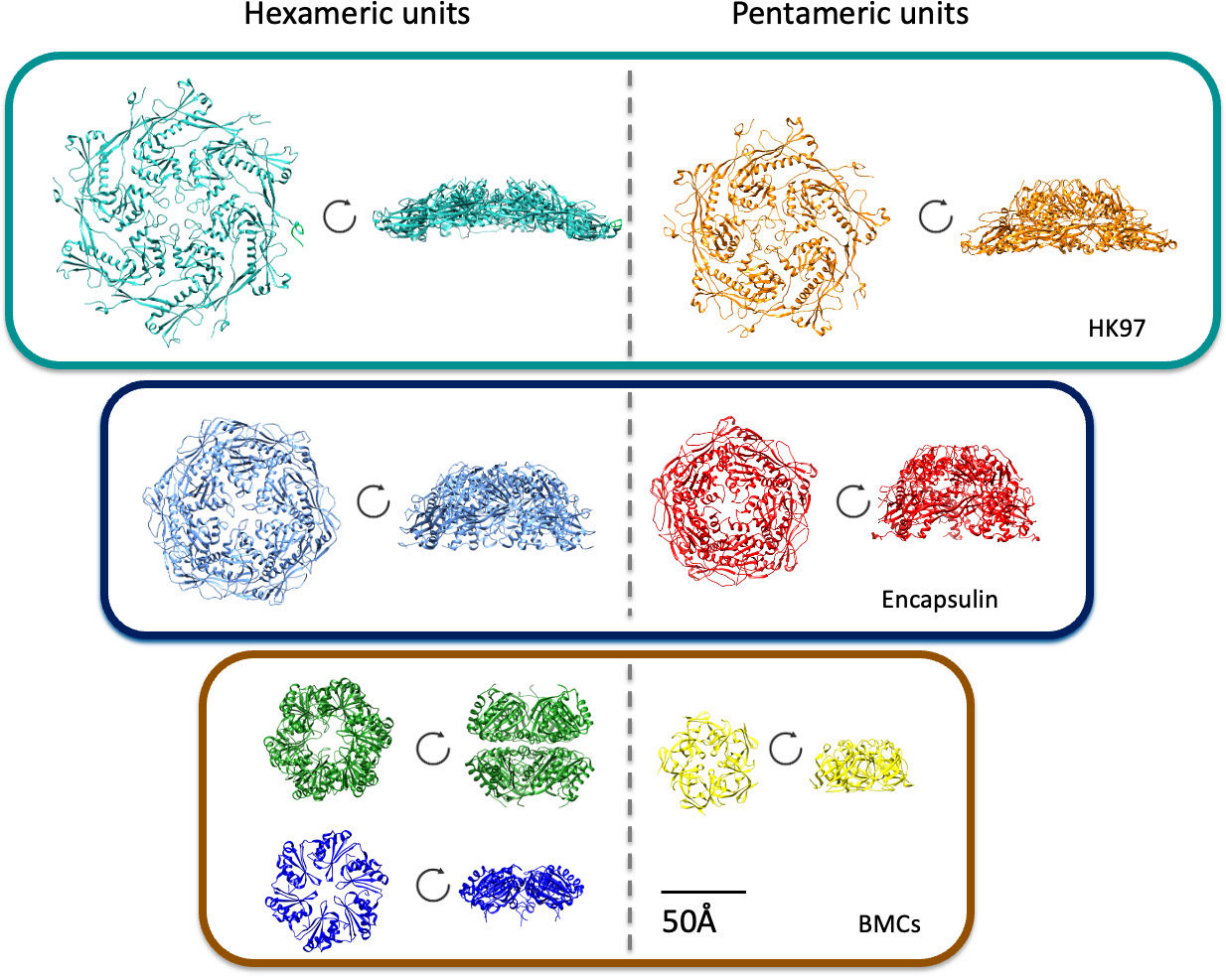
Tile subunits for viruses, encapsulins, and BMCs: Hexameric and pentameric grouping for HK97 (PDB 1OHG), *Myxococcus xanthus* EncA (PDB 8VJO), and *Haliangium ochraceum* microcompartment shells (PDB 5V74). Note, there are two types of hexameric building blocks for microcompartment shells: BMC-T (green) and BMC-H (blue). Top-down and side views are shown. All images are at the same scale (50 Å).

**Fig 3:**
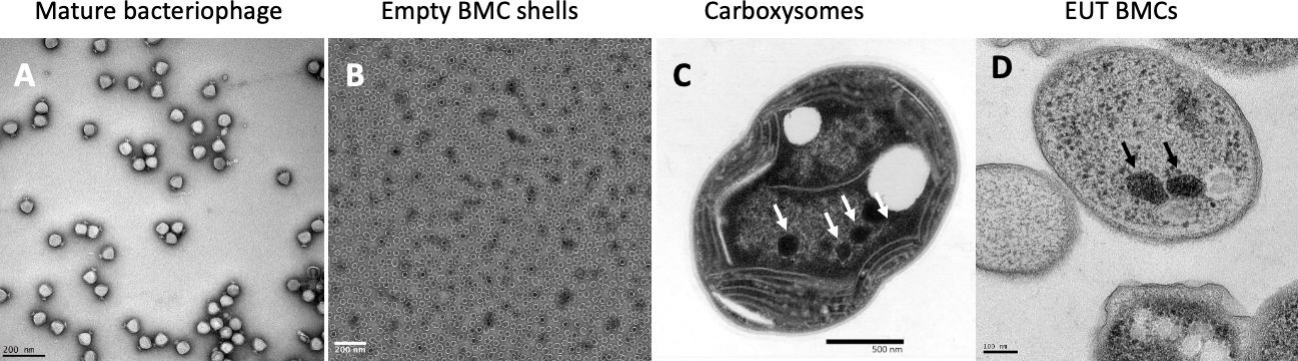
Bacteriophage, BMCs, and carboxysome morphologies: *in vivo* assembled particles. (**A**) A representative micrograph of negatively stained short-tailed podophage CUS-3. Image by Kristin Parent. (**B**) Negatively stained empty BMC shells produced by heterologous expression of *Haliangium ochraceum* shell proteins. Image courtesy of Tim Chiang, reproduced with permission. (**C**) Negatively stained thin section micrograph showing cyanobacterial carboxysomes. Image courtesy of Sarah Pacheco, reproduced with permission. (**D**) Negatively stained thin section micrograph showing EUT BMCs. Image courtesy of Doug Huseby, Rick Webb, and Cheryl Kerfeld, reproduced with permission.

In contrast, BMCs, which are organelles that compartmentalize segments of metabolic pathways in shells, are formed from hexagonal and pentagonal protein oligomers formed by distinct protein families. The Pfam 00936 domain forms hexameric (BMC-H proteins) or trimeric (BMC-T proteins) oligomers structurally comparable to hexons, and the Pfam 03319 domain (BMC-P proteins) that form pentameric oligomers (Fig. 2). These protein families are unrelated to any known viral proteins, but are shared among all functionally diverse BMCs. Moreover, unlike viruses and encapsulins, BMCs more frequently appear as polyhedra, rather than as icosahedra, are usually much larger (100–600 nm) (35–41), and all BMC shells are composed of multiple paralogous shell proteins. A survey of all functionally diverse BMC loci in genomic sequence data showed that, on average, shell chromosomal loci encode 1.7 BMC-P, 2.6 BMC-H, and 1.2 BMC-T proteins (10). This likely reflects a key functional attribute of BMC shells: selective permeability for the substrates and products of the encapsulated enzymatic reactions. This multiplicity, and that the composition of the shell may be responsive to environmental conditions, may contribute to the observed pleiomorphy of native BMCs (Fig. 3). However, when most BMC shell proteins are expressed or assembled together *in vitro*, in the absence of their protein cargo, they typically form regular icosahedra that are very homogeneous (Fig. 3) (42–47), structurally analogous to viruses. However, there are some exceptions to this rule (48, 49); this point will be discussed more in-depth in later sections.

### Assembly pathways: commonalities and deviations among shells

Smaller T number shells (ranging in complexity from T=1 to T=52 for viruses), encapsulins (T=1 to T=4), and empty BMC shells (T=4 to T=9) follow traditional groupings of hexons and pentons observed in classically described icosahedra (50). These organizations start to shift at larger sizes in viruses (e.g., giant viruses; T=>100), as they take on a different global organization formed from larger aggregates called pentasymmetrons and trisymmetrons (51). No such aggregate basic units are known for empty BMC shells or native BMCs (i.e., composed of protein cargo and shell).

While smaller T number icosahedral viruses all form from the same organization of hexon and penton building blocks, there is a distinct deviation in the order of assembly. Many icosahedral viruses typically assemble from a penton first. Rules governing icosahedral virus assembly have been derived from a number of well-studied groups. First, some viral capsids are formed exclusively from pentons, such as T=1 capsids, including animal viruses such as parvoviruses (52), fungal viruses, and plant viruses (53, 54). These penton-only structures suggest hexons are dispensable for initiating assembly. Additionally, the T=7 polyoma virus assembles from 72 pentameric subunits (55), indicating hexons are not necessarily needed to form larger, more complex icosahedra. Other evidence suggests that a wide array of viruses assemble first from pentameric vertices such as most, if not all, dsDNA phage and some eukaryotic viruses (herpes and adenovirus), ssDNA phage, and giant viruses (56–58). In many cases, this may result from the requirement of breaking strict icosahedral symmetry at a single unique vertex that can accommodate a portal or a stargate for dsDNA packaging and egress, and may also serve as the site of tail or tube attachment (59). However, not all icosahedral viruses begin assembly at a five-fold axis of symmetry. For example, phage MS2 assembly is proposed to initiate at a threefold symmetry site (60).

Interestingly, the penton-hexon interface can be a weak point in icosahedral shells. For phage P22, heat stress can cause the pentons to release (61, 62). Atomic force microscopy studies also show mechanical failure for herpesviruses is localized to the pentons as well (63). For empty BMC shells, incomplete occupancy of pentamers is well documented. This was first observed during the structure determination of an empty metabolosome shell. Crystals of 6.5 MDa empty recombinant BMC shell initially diffracted poorly. Expression of additional copies of the pentamer improved diffraction to 3.5 Å (43), leading to a model containing all of the distinct subunit interactions within an empty, icosahedral BMC shell. For native BMCs, fluorescence-based imaging methods for inventorying the components of the beta carboxysome indicate that pentamers are present in substoichiometric amounts (64, 65). This has been suggested to reflect dynamics of pentamer association/dissociation in native BMCs, which could allow for enzymatic core remodeling and repair and is proposed as a factor in the observed morphological heterogeneity (64, 65). Currently, little is known about the assembly pathway for empty BMC shells, but what we do know suggests it is likely not driven by pentamer-first kinetics, as pentons can be absent.

### *In vivo* assembly pathways among biological shells and departures from icosahedral symmetry

Initiation of assembly differs among naturally occurring biological polyhedra. Some viral capsids, such as all dsDNA-containing tailed bacteriophages and herpesviruses, assemble first into precursor structures called procapsids from interactions between the major capsid protein and the scaffolding proteins, which are largely helical proteins that assemble the major coat protein subunits via electrostatic interactions (66–74). Scaffolding proteins then either leave the shells intact (e.g., phage P22) or are cleaved (e.g., herpesviruses), and the peptide byproducts leave via pores in the hexons. A maturation event then typically occurs that results in expansion of the head volume to accommodate packaging of the genome by ATP-driven motors, most often terminases (18), but other motor types are observed as well, such as in phi29 (75). Similar morphogenesis pathways likely occur for some giant viruses that also contain portal complexes and active ATPase packaging proteins (76).

In addition to the highly specific interactions between scaffolding proteins and the major coat proteins, some viruses also selectively package other phage-encoded proteins that remain inside the viral shell during maturation. Some examples are the so-called “E-proteins,” or ejection proteins in phages P22 (77), DEV (78), and the H protein in phiX174 (79). These encapsidated proteins are used during the infection step; they are jettisoned from inside the capsid to form a tubular structure that helps transfer the genome from the phage across the host cell membrane into the cytoplasm. Another example of selectively packaged proteins in viral capsids includes virally encoded polymerases such as seen in phage N4 (80) as well as Hepatitis B Virus (81), rotaviruses (82), and reoviruses (83). Lastly, some Mimivirus-like giant viruses selectively package a large number of different proteins (~50–80 distinct proteins) into an extra membrane sac which is encapsidated in the virion, and is also likely used during the initial stages of infection (84). Alternatively, many icosahedral viruses can assemble directly around their genomes, especially viruses with single-stranded genomes (85), for example, ssRNA phages like MS2 (60), plant viruses such as CCMV (86), or animal viruses such as picornoviruses (87, 88). Regardless of whether it is scaffolding protein-mediated or genome-mediated assembly, the end state for all known mature virions is that the majority of packaged cargo is genetic material, be it RNA, DNA, or a mixture of both, with a minority of the cargo (34) including proteins.

In addition to classical wet lab experimentation, computational approaches are also being applied to study assembly of isometric viral shells (89, 90), as well as less regularly shaped (e.g., human immunodeficiency virus [91] and others [92]), and much larger polyhedra (e.g., giant viruses [93]). The following is a brief summation of the general principles learned that govern icosahedral assembly for a variety of examples. Assembly of icosahedral cages can either be by (i) the addition of monomeric subunits, one at a time (e.g., phage P22 [94]), (ii) by preformed tiles already assembled into hexon and penton organizations (e.g., phage HK97 [95]), or (iii) in smaller viruses, assembly can be observed from dimers as in hepatitis B virus (HBV) (96). Both traditional experimentation and computational approaches converge to similar themes: these assembly pathways are generally high fidelity and occur on similar kinetic time scales (85, 97). Time scales of assembly are linked tightly to the biology. As one example, for bacteriophages, assembly occurs within minutes at biological temperatures as the doubling time of the host is usually on the order of 20–30 minutes. Similarly, assembly of encapsulins is observed to take ~15–30 minutes *in vitro* (98). However, our understanding of the influence of kinetics on capsid assembly is not yet complete. Kinetic traps can form if assembly rates, especially involving nucleation, are too high, as too many starting places form and free subunits become rapidly depleted (99), resulting in incomplete particles. By contrast, a more recent study suggested that faster assembly times are beneficial, as they can lead to fewer aberrant particles by skipping over highly stable pentameric kinetic traps, as shown with human papillomavirus (HPV) (100). Assembly environment matters: coarse-grained molecular dynamics simulations show that biomolecular condensates can enhance assembly rates and robustness, and this might explain the importance of viral factories (101). Perhaps if slow and “correct” nucleation is achieved, coupled with faster elongation, kinetics can improve viral capsid yields.

Once formed, viral icosahedral shells tend to be highly stable. And from an assembly perspective, relatively weak protein:protein interactions build to form very stable icosahedra products (102, 103). Yet, dissociation of viral subunits can also be observed on experimental time scales, indicating these are not inert, irreversible assemblies (104, 105). In addition, many viruses need to dissociate or disrupt their icosahedral capsids *in vivo* as part of the natural infection processes. Often, overcoming this thermodynamic barrier to disrupting the icosahedral shell requires perturbation. Some examples can be from interactions with cellular factors, such as receptor proteins (106), interactions with the nuclear pore complex (107), changes in pH (84), and others, as well as interactions with abiotic factors, such as binding to drug molecules (108), etc. Disassembly pathways have been notoriously difficult to study experimentally; this is an emerging area for computational approaches such as molecular dynamics (109).

In contrast, encapsulins and BMCs exclusively compartmentalize proteinaceous cargo, rather than nucleic acids. BMCs are metabolically diverse—there are over 60 functionally distinct types encapsulating a diverse array of enzymes and type-specific scaffolding proteins (10, 110). Encapsulins, on the other hand, are more limited in the types and amounts of cargo they encapsulate via targeting domains/peptides (28, 31, 111). Encapsulin assembly is more similar to virus assembly than to BMC assembly. Phage and encapsulin building blocks have homologous folds (Fig. 1). The assembly process of encapsulins is also highly specific. It is usually driven by the products of a two-gene system, including a shell protein and a cargo protein. Encapsulin assembly pathways can occur via one of two “modes.” Either C-terminal peptides drive specific cargo encapsulation, or N-terminal domains regulate assembly (112, 113). Recent work using cryo-EM studies has revealed encapsulin assembly intermediates both *in vivo* and *in vitro* and suggests assembly is driven by pairwise addition of monomers (114).

Unlike both viruses and encapsulins, departure from strict icosahedral symmetry is the rule, not the exception, for native BMC systems (Fig. 3) (35–41). Furthermore, despite their structurally analogous shell protein building blocks with their potential to self-assemble into icosahedra (Fig. 2), unlike viruses, assembly of BMCs is strongly influenced by cargo. Theoretical studies have underscored the important role of the encapsulated proteins in dictating BMC size and morphology (115–118) through both kinetic and thermodynamic influences.

Given the observed heterogeneity of BMC polyhedra in cells (Fig. 3), diverse pathways for their assembly are expected. In the first BMC assembly pathway to be experimentally deduced, for the beta carboxysome (Fig. 3C), nucleation of the enzymatic core is the initial step, followed by incorporation of the shell. In this BMC, the central cargo enzyme ribulose-1,5-bisphosphate carboxylase/oxygenase (RuBisCo) is composed of eight small (RbcS) and eight large (RbcL) subunits. It is packaged with a gamma carbonic anhydrase, CcmM, which, in addition to its carbonic anhydrase domain, contains multiple copies of RbcS-like domains, which provide interaction sites with RuBisCo. A third protein, CcmN, contains gamma carbonic anhydrase-like repeats, a variable length, poorly conserved, disordered linker region, and a C-terminal encapsulation peptide (119), shown to interact with the carboxysome shell proteins (115). This type of assembly pathway, involving both enzymes and proteins with domains or regions that mimic the enzyme components to function in scaffolding (115, 117), is likely to be widespread among functionally diverse BMCs. Domain mimics are known in several BMC types (10, 120), and encapsulation peptides are found in the majority of BMCs (119, 121). Indeed, encapsulation peptides have long been known to cause aggregation of BMC enzymes (122–124) and now are emerging as part of a general strategy for biomolecular condensation with applications in biotechnology (125).

In contrast, the alpha carboxysome, which is evolutionarily distinct from the beta carboxysome (126), appears to have distinct assembly modes; both potential for a “shell-first” pathway (127) or concomitant assembly of shell and core proteins (128, 129). Notably, the morphology and size of alpha carboxysomes tend to be more regular and more frequently icosahedral; this may be related to more complete capping of the vertices by the BMC-P paralogs (130). Similar flexibility in the assembly modes of the propanediol metabolosome (PDU BMCs) was recently described (131), as well as the observation that specific extensions of some shell proteins play a role in the interactions between shell and cargo (131, 132). Likewise, similar to the beta carboxysome and other characterized BMC cores (133, 134), cargo enzymes form complexes that likely enhance catalytic efficiency. Assembly of the ethanolamine (“EUT”) metabolosome (Fig. 3B) provides a third variation among BMC assembly themes; it is initiated by shell formation; however, distinct aggregates of both the shell and the core enzymes can form as stages in assembly (135).

### Convergences of capsids and BMC shells in pleiomorphic space

Although relatively less frequently observed, viral capsids departing from symmetric icosahedral symmetry can occur by a number of means and still result in the formation of functional shells. Some examples include altered distribution of pentamers. For example, cones of various sizes and overall morphology can be seen in human immunodeficiency virus, HIV-1 by cryo-electron tomography (136). Interestingly, an empty BMC shell with a similar nanocone morphology is found among icosahedral shells and nanotubes formed by heterologous expression of the six distinct shell proteins (4 BMC-H, 1 BMC-T, and 1 BMC-P) from a purple bacterial BMC (49). In addition, partially capped “whiffle ball” BMC shells can form, and these demonstrate increased permeability relative to fully capped shells (137). Some natural BMC systems, such as glycyl radical enzyme-associated microcompartments (“GRM”), have enormous shape variation (36), and others like HO are more uniform. It is unknown yet whether or not the polyhedral shape of *in vivo* assembled shells is critical for their function. However, observational studies show that in HO shells, the less uniform shells (*in vivo-*assembled) have similar catalytic activity of packaged cargo compared with the *in vitro* assembled shells (138). Combined, these data suggest uniform morphology shells may be fully functional, and that polyhedral variance is likely not by design or necessity.

In viruses, off-pathway assemblies can also occur that drive the formation of non-functional forms. Phage MS2 can naturally assemble into a mixture of forms, including functional particles as well as oblong shapes, both *in silico* (139) and *in vivo* (140), by altered interactions with the RNA genome. Mixed populations of icosahedra containing smaller or larger triangulation numbers can occur natively such as the presence of both T=3 and T=4 particles in hepatitis B virus infections (141, 142). Deviations in terms of frequency of “incorrectly” shaped or sized viral particles within populations can arise due to mutations or deletions in genes of regulating proteins, such as scaffolding proteins. Both mutations and deletions can give rise to altered virion types at an increased frequency compared to native infections. Commonly observed off-pathway assemblies have been studied extensively in bacteriophage P22. For example, deletion of scaffolding protein results in both T=7 native particles, as well as smaller T=4 particles (143). Mutations in either the coat protein (144) or deletions in the scaffolding protein (145) can also give rise to “spirals,” where the pentons are located inappropriately, and the shells are unable to form completely closed icosahedra. In addition, mutations can also cause non-functional particle types such as sheets and tubes, which can form from relatively conservative, single amino acid capsid protein substitutions (144, 146, 147). For BMC systems, sheets and tubes are readily assembled by diverse shell proteins when assembled without cargo and out of their native context (42–45, 47, 49, 148–153).

## SUMMARY

Why is the icosahedron so prevalent in nature? Evolutionarily very diverse protein folds (Fig. 1) seem to converge to self-assemble into the same structure (icosahedron) for two main reasons: stability and efficiency. An icosahedron requires a limited number of genes and protein building blocks.

For viruses, smaller capsids hold tiny genomes, and the same capsid geometry can expand to accommodate a massive amount of genetic material—up to 2.5 Mbp for giant viruses. Pentons are needed to provide the curvature to preferentially form a sphere over a tube and to complete the shells, but wiffle balls (viral shells without pentons) are stable too. There are a limited number of polyhedra formed from identical building blocks (cube, tetrahedron, and octahedron) that are scalable (icosahedral and dodecahedral). Icosahedral structures are ideally suited for biological assemblies given their increased rotational symmetry over other forms, and this optimizes internal volumes needed for genome packaging efficiency, particularly important for larger T number viruses (154). BMC shell proteins in the absence of their cargo readily assemble into icosahedra but are more commonly polyhedral in their native context. In bacteria, BMC shells function in concert with the requirements of the encapsulated enzymes; the shells not only protect the enzymes but also serve as conduits for their substrates and products. The multiplicity of enzymes and the structural redundancy arising from the use of paralogous hexagonal tiles (BMC-H and BMC-T proteins) and pentagonal tiles (BMC-P) are likely related to the frequent observations of various sizes and polyhedral morphologies of BMCs in cells. Despite being composed of structurally analogous building blocks, cargo-dictated function of viral capsids and BMC shells likely underlies their contrasting adherence to and departure from strict icosahedral form.
